# An Evidence-Based Multidisciplinary Practice Guideline to Reduce the Workload due to Lifting for Preventing Work-Related Low Back Pain

**DOI:** 10.1186/2052-4374-26-16

**Published:** 2014-06-24

**Authors:** P Paul FM Kuijer, Jos HAM Verbeek, Bart Visser, Leo AM Elders, Nico Van Roden, Marion ER Van den Wittenboer, Marian Lebbink, Alex Burdorf, Carel TJ Hulshof

**Affiliations:** 1 Netherlands Center for Occupational Diseases, Coronel Institute of Occupational Health, Academic Medical Center, University of Amsterdam, PO Box 22700, 1100 DE Amsterdam, The Netherlands; 2 Centre of Excellence, the Netherlands Society of Occupational Medicine (NVAB), Utrecht, the Netherlands; 3 Finnish Institute of Occupational Health, Kuopio, Finland; 4 Amsterdam School of Health Professions, Amsterdam University of Applied Sciences, Amsterdam, the Netherlands; 5 Dutch Society of Safety Science, Eindhoven, the Netherlands; 6 Professional Association of Work and Organizational Experts, Eindhoven, the Netherlands; 7 Department of Public Health, Erasmus MC, Rotterdam, the Netherlands

**Keywords:** Back pain, Interventions, Occupational health care, Prevention, Surveillance, Practice guideline, Lifting

## Abstract

We developed an evidence-based practice guideline to support occupational safety and health (OSH) professionals in assessing the risk due to lifting and in selecting effective preventive measures for low back pain (LBP) in the Netherlands. The guideline was developed at the request of the Dutch government by a project team of experts and OSH professionals in lifting and work-related LBP. The recommendations for risk assessment were based on the quality of instruments to assess the risk on LBP due to lifting. Recommendations for interventions were based on a systematic review of the effects of worker- and work directed interventions to reduce back load due to lifting. The quality of the evidence was rated as strong (A), moderate (B), limited (C) or based on consensus (D). Finally, eight experts and twenty-four OSH professionals commented on and evaluated the content and the feasibility of the preliminary guideline. For risk assessment we recommend loads heavier than 25 kg always to be considered a risk for LBP while loads less than 3 kg do not pose a risk. For loads between 3–25 kg, risk assessment shall be performed using the Manual handling Assessment Charts (MAC)-Tool or National Institute for Occupational Safety and Health (NIOSH) lifting equation. Effective work oriented interventions are patient lifting devices (Level A) and lifting devices for goods (Level C), optimizing working height (Level A) and reducing load mass (Level C). Ineffective work oriented preventive measures are regulations to ban lifting without proper alternatives (Level D). We do not recommend worker-oriented interventions but consider personal lift assist devices as promising (Level C). Ineffective worker-oriented preventive measures are training in lifting technique (Level A), use of back-belts (Level A) and pre-employment medical examinations (Level A). This multidisciplinary evidence-based practice guideline gives clear criteria whether an employee is at risk for LBP while lifting and provides an easy-reference for (in)effective risk reduction measures based on scientific evidence, experience, and consensus among OSH experts and practitioners.

## Introduction

Lifting is an activity that is common during work. In a European study on working conditions, 35% of the employees reported manual lifting or carrying of loads on a regular basis. Despite automation, European workers are equally exposed to lifting and carrying as they did 10 years ago [1]. In the Dutch National Survey on Working Conditions 2010, 16% of the employees are convinced that preventive measures at the workplace are necessary to reduce the physical workload due to lifting [2]. In the Netherlands, manual lifting is performed most frequently in construction, transport and industry. In these sectors, more than 20% of the employees think that preventive measures are needed to reduce the physical workload [2].

In the last ten years, comprehensive literature overviews concerning health risks, especially low back pain (LBP), due to lifting have been published [3-8]. Despite debate regarding the methodological errors and biases of these overviews [9-14], there is evidence for a relationship between lifting and LBP. The Health Council of the Netherlands calculated that every year, 13 of every 100 employees report a new episode of LBP [15]. Lifting 10 kg regularly at work would result in 1.4 extra cases of LBP for these 100 employees per year. Lifting 23 kg would result in 3.3 extra cases of LBP.

LBP - after flu – is the second most important reason for sick leave and is responsible for 15% of the annual number of sick leave days in the Netherlands [16]. Of these employees on sick leave, 21% indicated that their work is the main cause of these symptoms and 32% states that their work is partly the cause [16]. Given the multifactorial nature of LBP, in their duty of notifying occupational diseases, occupational physicians in the Netherlands are supported by an evidence-based diagnostic guideline for deciding on the work-related nature of LBP [17-19]. In 2013, occupational physicians in the Netherlands reported 505 cases of back pain as occupational diseases. This is 26% of the total number of reported occupational musculoskeletal disorders. Similar results have been reported for Korea [20] or the United Kingdom [21]. Worldwide, 37% of adult cases of LBP are attributed to occupation, with an estimated annual loss of 818,000 disability-adjusted life years worldwide [22].

Although knowledge is gained about the possible work-related causes and prevention of LBP, we seem to make little progress in preventing this important work-related complaint. This slow progress is not for want of trying [23]. For example, in 2007 the European Agency for Safety and Health at Work organized a major campaign, “Lighten the Load – How to prevent Musculoskeletal Disorders (MSDs)” and the National Institute of Occupational Safety and Health (NIOSH) in the United States of America specifically identified musculoskeletal disorders as a major focus in their National Occupational Research Agenda. Wells [23] formulated six explanations or ‘weakest links’ in terms of research questions why so little progress was made. Three of these questions were: 1) how good are our MSD risk factors, 2) how effective and informative are current workplace MSD assessment approaches, and 3) how effective are the recommended interventions in actually reducing MSDs in the workplace?

In order to answer these three questions in the best possible way, the Dutch Government granted a project on the development of an evidence-based practice guideline to support occupational safety and health (OSH) professionals in the Netherlands in their decision making whether lifting at work can be considered a risk factor for LBP and, consequently, which interventions can be recommended. This practice guideline should be based on the best available scientific evidence, integrated with the expertise of OSH professionals and taking into account the values and preferences of employers and employees [24,25]. This paper describes the development and content of this practice guideline.

### Practice guideline: scientific evidence and professional expertise

The practice guideline was developed by a project team of OSH experts and practitioners and is based on:

1) an evaluation of the quality of methods for risk assessment of workplaces that involve lifting;

2) the results of a systematic review of the effects of interventions for reducing biomechanical loading of the back due to lifting;

3) an evaluation of the feasibility of the draft guideline by external peer reviewers and a practice test on feasibility of the draft by OSH professionals.

This paper provides an overview of the main findings and recommendations. For detailed information we refer to the Dutch background document of the multidisciplinary practice guideline (In Dutch: nvab.artsennet.nl/Richtlijnen/NVABrichtlijnen-en-procedurele-leidraden/Richtlijn-Tillen.htm)*.*

### Expert meetings

The project team consisted of nine persons, experts and practitioners in the development and implementations of clinical practice guidelines and/or lifting and work-related LBP. All members are authors of this paper. The group met nine times in various compositions during a period of 12 months to discuss all relevant documents, evidence reports, and specific recommendations. Consensus was reached on all decisions regarding evidence reports and the specific recommendations.

## Review

### Methods to assess the risk of LBP due to lifting

A systematic search was performed in OVID SP, and the same time in Embase (1974-November 8^th^ 2011) and Medline (1945-November 8^th^ 2011) for systematic reviews regarding the quality of risk assessments methods for physical workload. The search terms are listed in Table 1.

**Table 1:** Search terms for systematic reviews regarding the clinimetric quality of assessments methods for workload due to lifting

**Key term**	**Search terms**
Systematic review	(meta-analysis/or meta-analysis.pt. or meta-analysis.ti,ab. or review.pt. or review.ti,ab.) not ((letter or editorial or comment).pt. not (animals/not humans/))
Clinimetric quality assessment methods	(responsiveness$ or reliability or validity).ti,ab. or “Sensitivity and Specificity”/or “Reproducibility of Results”/or reproducibility.ti,ab. or agreement.ti,ab. or psychometric$.ti,ab. or (gold adj standard).ti,ab. or (content adj validity).ti,ab. or (minimal adj clinical adj difference).ti,ab. or (clinical adj change).ti,ab. or (important adj change).ti,ab. or (important adj difference).ti,ab.
Lifting, work load	((lift$ or (manual adj material adj handl$) or (handling adj load$) or (handling adj1 patient$) or (exposure adj measurement$) or (physical adj work adj load) or (physical adj work) or (physical adj workload) or (physical adj work adj demand$) or (biomechanical adj exposure$) or (mechanical adj exposure$) or (mechanical adj demand$)) not (face adj lift$)).ti,ab.

Papers were included if they met the following inclusion criteria:

•The paper is a systematic review that describes risk assessment methods for the physical workload due to lifting of employee’s;

•The assessment methods described can be used by OSH professionals in practice like observations, questionnaires, task analyses, interviews, diaries, or self-reports;

•The paper describes the quality of the assessment methods in terms of validity or reproducibility.

If title and abstract did not provide enough information to decide whether the inclusion criteria were met, the full paper was checked. Next, the inclusion criteria were applied to the full paper. Finally, the references of the included papers were also checked for other potentially relevant papers.

The search strategy resulted in 176 references, and after duplication 121 remained. The full text was read of 17 papers of which nine fulfilled the inclusion criteria. Checking the references resulted in five extra papers. We performed critical appraisal of all included papers using the criteria of the Dutch Institute for Healthcare Improvement with the following levels of evidence: A (strong), B (moderate), C (limited) and D (consensus) [26].

### Risk assessment approach

**Figure 1:**
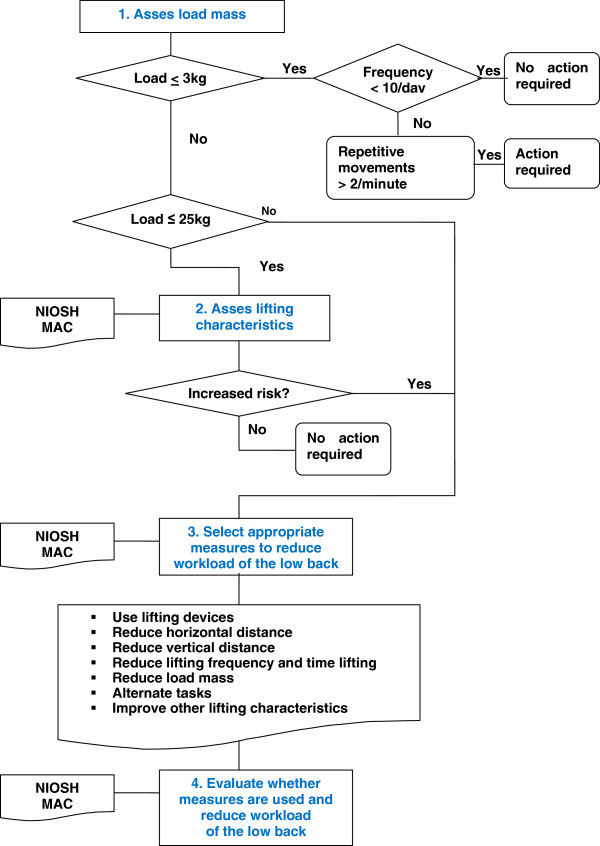
Flow chart consisting of four steps to assess the risk of manual lifting to prevent work-related low back pain (NIOSH: National Institute for Occupational Safety and Health lifting equation, MAC = Manual handling Assessment Charts-tool).

The project team selected observation methods above self-reports to assess the risks of physical work load due to lifting. The main reason was that observation methods used by trained OSH professionals would result in more valid assessments [27]. Takala et al. [28] described seven observation methods of which the NIOSH lifting equation was the most renowned. Four of these methods were not tested for validity or reliability. In addition to the NIOSH lifting equation, the Manual handling Assessment Charts (MAC)-Tool and the Washington State Ergonomic Checklist for Manual Handling remained. The latter was developed to evaluate only high risk work situations and was therefore excluded. Besides the NIOSH lifting equation and the MAC-Tool, also the Key Indicator Method (KIM) assesses the risk due to lifting. The use of the KIM is in Europe strongly supported by the Senior Labour Inspectorate Committee and by the European Agency for Safety and Health (http://osha.europa.eu/en/topics/msds/slic/handlingloads/19.htm). This method was not described in the review by Takala et al. [28], because no studies were published in peer-reviewed international journals regarding its clinimetric properties. Moreover, the results of the KIM appear not to be in line with epidemiological evidence on risk factors: 40 kg is taken as the maximum acceptable load [29]. Therefore, we recommend the NIOSH lifting equation [30] and the MAC-Tool [31] to assess the risks of lifting situations (Level A) [28] (Figure 1). In addition, it was agreed upon that lifting loads less than 3 kg was not considered a risk for LBP if the lifting frequency was less than 10 times a day (Figure 1). If objects of less than 3 kg were manually handled for more than 2 times a minute, an assessment method for upper extremity complaints should be used. Loads heavier than 25 kg regardless of the frequency were considered to be a risk factor for LBP (Level D) [3,17,30,32].

### Interventions

We used both back pain and back load as outcomes measures to decide if interventions were effective. For low back load we used compression forces, electromyography (EMG) of trunk muscles, trunk postures or time lifting as valid measures of load. The reasons were threefold. First, a recent health impact assessment on the effect of lifting devices demonstrated that the impact of this intervention could only be evaluated properly by estimating the reduction in exposure to lifting activities and, subsequently, determine the influence of this reduction in exposure on the decrease in occurrence in LBP [33]. Second, promising interventions to reduce the risk of LBP are often evaluated on its efficacy in practice using outcome measures like low back loads. Third, although the exact aetiology for LBP is still unknown, the assumption is that low back loading is an independent contributing factor [14].

In our systematic review of the effects of interventions, we included any study that evaluated the effect on low back load or LBP due to lifting at work in a field experiment. We did not include laboratory experiments because it is difficult to translate such results to practice. We made a distinction between measures directed at the worker like the use of back-belts or directed at the work situation like the use of lifting devices.

We performed a systematic search with OVID SP in Embase (1974-November 8^th^ 2011) and Medline (1945-November 8^th^ 2011) for reviews and primary studies regarding the efficacy of interventions to reduce low back load or LBP due to lifting. The search terms are listed in Table 2.

**Table 2:** Search terms for review and primary studies on the effectiveness of interventions to reduce the low back load due to lifting at the work

**Key term**	**Search terms**
Intervention, work	((train$ or advi$ or educa$ or inform$ or guid$ or promot$) adj3 lift$).ti,ab OR ((lift$ adj3 policy) or zero-lift$ or no-lift$).ti,ab OR ((lift$ or material handling or (patient$ adj (transfer or lift or handling))) adj3 (aid$ or device$ or equipment or system$)).ti,ab OR (hoist$ or winch or ((table or platform or drum) adj3 lift$) or trolle$ or “fork-lift truck” or (yoke adj5 lift$) or exo-skeleton).ti,ab OR ((sling adj3 (lift$ or transfer$ or handl$)) or (glid$ adj3 sheet$) or ((back or lift$) adj3 belt$)).ti,ab OR ((workplace or ergonom$) adj3 (accommodation$ or change$ or improve$ or intervention$)).ti,ab
Low back	(back or trunk or body).mp

After omitting duplicate papers, the search strategy resulted in 1089 papers. Twelve reviews and 51 primary studies fulfilled our inclusion criteria. The included papers of primary studies were performed in building and construction, in industry and transport, in agriculture and fishing and in health care.

### Worker-directed interventions

#### Training and advice for optimizing lifting posture and movement

A systematic review of biomechanical studies [34] showed that no differences were found between the so called stoop or squat lifts, unless the handled object could pass between the legs. Lavender et al. [35] determined the degree to which a new behaviour-based lift training program reduced the low back load in distribution centre jobs that require repetitive lifting. A total of 2144 employees in 19 distribution centres were randomized into either the LiftTrainer program or a video control group. In the LiftTrainer program, participants were individually trained in up to 5, 30-minute sessions while instrumented with motion capture sensors to quantify the L5/S1 moments. The magnitude of this moment vector was used to adjust the pitch of a tone, the biofeedback signal, heard by the participant in such way that the higher the instantaneous spine moment magnitude, the higher the pitch of the tone. The decrements in the forward bending moments started off small, less than 5% in the first session but exceeded 10% in the final two sessions. Daltroy et al. [36] evaluated an educational program designed to reduce low back injuries. Physical therapists taught three hours of class sessions, including knowledge, skills, and individual work station assessment, to small groups of workers and supervisors, with reinforcement every 6 months afterward. At 2 1/2 years, a random sample of 209 workers was surveyed for program impact on intermediate outcomes. No significant improvements in behaviours associated with low back loading were observed. From these studies, we concluded that it is theoretically possible that training and advice reduce the load of the low back in the order of 5 to 10%, but that it is unlikely that this will be achieved and uphold in practice. This finding is in line with a Cochrane review on the effect of manual material handling advice and assistive devices for preventing back pain that did not find a considerable effect on the occurrence of back pain [37-39] (Level A, Table 3).

**Table 3:** The recommendations including the level of evidence (A strong, B moderate, C limited and D consensus) of the multidisciplinary practice guideline for preventive measures directed at the worker and at work based on studies with outcomes in terms of low back pain and/or low back load

**Preventive measure**	**Recommendation**	**Evidence**
**Worker**		
Training and advice for optimizing lifting posture and movement	‘It is theoretically possible that training and advice reduce the load of the low back in the order of 5 to 10%, but that it is unlikely that this will be achieved and uphold in practice. This recommendation is also in line with the Cochrane reviews on manual material handling advice and assistive devices for preventing and treating back pain in workers.’	A
Pre-employment medical examination	‘There is conflicting evidence in the two studies regarding the effect of a pre-employment examination that included a physical capacity evaluation on LBP. Due to the high rejection rate of candidates, a pre-employment medical examination is not recommended to reduce the risk of LBP.’	A
Back-belts	‘Back-belts are not more effective than no intervention or training in preventing low-back pain, and conflicting evidence whether lumbar supports were effective supplements to other preventive interventions. Therefore, the use back-belts as a personal protective device was not recommend.’	A
Personal lift assist devices	‘Personal lift assist devices are promising interventions in reducing the load on the low back. However, more research is needed to evaluate the effects on the longer term.’	C
**Work**		
*Eliminate manual lifting*		
Lifting devices patients	‘Lifting devices for patients are able to overcome manual lifting, although low back loading still occurs due to bending postures and the time it takes to prepare the patient for a transfer. Therefore in each setting a careful consideration has to be made. In addition, overhead or ceiling lifts are preferable above floor lifts.’	A
Lifting devices objects	‘The core group and project team concluded that case studies on the efficacy of lifting devices in construction, automotive industry and also fishing and agriculture show that in general the use of these devices reduce the low back load. However, this is not true for all tasks performed. A hindering factor is the increase in production time. Therefore in each setting a careful consideration has to be made.’	C
Production methods	‘A change in production methods for instance from manual lifting to pushing and pulling might result in a strong reduction of the low back load.’	C
*Improve lifting situation*		
Weight of object	‘A reduction in weight of the object does not always result in a reduction of the load of the low back due to a possible increase in exposure time or frequency or due to unfavourable characteristics of the load lifted.’	C
Vertical lifting distance	‘Aides to reduce the vertical lifting distance like scissor lifts or a scaffolding console can reduce the load on the low back considerable.’	B
Horizontal lifting distance and sliding friction	‘Aids to reduce the horizontal lifting distance or friction in patients transfers or while lifting objects like bridgeboards, rods, gliding sheets of rolling floors can reduce the load on the low back.’	A
Contact factor	‘Lifting belts for a better handling of patients contribute to a reduction of the low back loading while lifting.’	C
*Organisational factors*		
Lifting teams	‘Well-staffed lifting teams of specifically trained and equipped employees reduce the number of patient lifts that other colleagues had to perform without increasing the number of low back complaints in these lifting teams.’	B
Team lifting	‘Team lifting compared to one or two persons lifts does not result in an increased risk for low back pain.’	C
Regulations	‘The prohibition of manual lifting does only result in a reduction of low back loading if effective and efficient alternatives are available.’	D

### Pre-employment medical examination

Mahmud et al. [40] reported in their Cochrane review two controlled studies that evaluated the effect of pre-employment medical examinations versus no pre-employment examination on LBP among workers that frequently perform lifting tasks. One study found that employees who received a pre-employment examination that included a functional capacity evaluation were less likely to report LBP after 7.4 months follow-up compared to those who received a pre-employment examination. The rejection rate in this study was not known. In contrast, the other study showed neither evidence of an immediate effect nor of a long term effect over the course of 10 ½ years. The rejection rate in this study doubled after the introduction of the functional capacity evaluation. Therefore, we concluded that there is conflicting evidence in the two studies regarding the effect of a pre-employment examination that included a physical capacity evaluation on LBP. Due to the high rejection rate of candidates, a pre-employment medical examination is not recommended to reduce the risk of LBP (Level A, Table 3).

### Back-belts

Van Duijvenbode et al. [41] reviewed the effects of lumbar supports for prevention of LBP. Seven studies with 14,437 participants were included in their updated review. There was evidence that lumbar supports were not more effective than no intervention or training in preventing low-back pain, and conflicting evidence whether lumbar supports were effective supplements to other preventive interventions. We recommend not to use back-belts as a personal protective device to reduce the risk of LBP (Level A, Table 3).

### Personal lift assist devices

Personal lift assist devices are externally worn body devices that are developed to support the body and thereby reducing the low back load. The positive results in several laboratory experiments were also found in a randomized field experiment among 10 assembly workers in the automotive industry who performed an on-line assembly process requiring forward bending and static holding [42]. Because there was only one small field experiment we did not recommend this as an intervention but we concluded that personal lift assist devices may be promising interventions in reducing the load on the back (Level C, Table 3).

### Work-directed interventions

#### Eliminate manual lifting

Manual lifting can be overcome by introducing lifting devices or by introducing other production methods. A distinction is made between lifting patients in health care and lifting loads in construction, agriculture or automotive industry. For patients, Santaguida et al. [43] assessed the spinal loading while performing a bed to chair transfer comparing overhead and floor powered lifting devices. Overhead lifting devices were shown to have lower spinal loads during the transport phases and were preferred by the nurses. We concluded that lifting devices are able to overcome manual lifting, although low back loading still occurs due to bending postures and the time it takes to prepare the patient for a transfer. For this reason, in each setting a careful consideration has to be made. In addition overhead or ceiling lifts are preferable above floor lifts (Level A, Table 3) [43-47].

For lifting objects, fewer studies have been performed and also with shorter follow up periods compared to lifting devices for patients. We concluded that in most case studies on the efficacy of lifting devices in construction, automotive industry and also fishing and agriculture showed that in general the use of these devices reduce the low back load . However, this was not the case for all tasks performed. A drawback of most lifting devices is the increase in production time. We recommend that in each setting a careful consideration has to be made between benefits and drawbacks (Level C, Table 3) [48-54].

An example of a study on other production methods, is a study among waste collectors using bags, two-wheeled containers or four-wheeled containers [55]. On the basis of the frequency and magnitude of spinal forces it was concluded that the mini-containers should be preferred to the bags and if four-wheeled containers are transported by two persons instead of one and the kerbs are removed this might also be a favourable method. We concluded that a change in production methods for instance from manual lifting to pushing and pulling can result in a strong reduction of the low back load (Level C, Table 3) [55].

### Improve lifting situation

Reduction of the weight of the object (Level C), the vertical lifting distance (Level B), the horizontal lifting distance and sliding friction (Level A), and a better contact factor (Level C), can reduce the load on the low back. This is in line with the risk factors from the NIOSH lifting equation [30] and the MAC-Tool [31]. For all these factors, studies were found that showed that that preventive measures that optimize these factors can reduce the load on the low back (Table 3). A scaffolding console to adjust the working height of the storage of materials resulted in a significant reduction of the frequency and duration of trunk flexion (>60 degrees) by 79% and 52% respectively, compared with bricks set out on the ground floor [56]. Hignett [57] found evidence in their review for the provision of a minimal set of equipment for moving and handling patients. The use of a rolling floor build inside the cargo space of a truck decreased the frequency of lifting and setting down goods by 24%, and decreased the frequency of handling goods below knee level by 79% [58].

### Organizational factors

We also considered the evidence for the organizational factors lifting teams, team lifting, job content and duration and regulations (Table 3). Well-staffed and equipped lifting teams may perform the majority of high risk lifts and transfers on shifts in which they operate and can reduce the number of low back injuries [59-62] (Level B, Table 3). Although team lifting increased the variability of the lifts, team lifting did not result in larger maximum peak lumbar compression forces compared to one or two persons lifts in ironworkers [62-64] (Level C, Table 3). Finally, the project team was of the opinion that regulation that prohibits manual lifting will only work when effective and efficient alternatives are available (Level D, Table 3).

### Feasibility study

Eight experts and twenty-four OSH professionals, with at least two of all the participating professional associations commented on and evaluated the content and the feasibility of the preliminary guideline. Thirteen topics were evaluated such as whether the goal of the practice guideline was clearly formulated, whether the procedure to assess the risk of lifting was feasible in practice, and whether the recommendations for interventions were clearly formulated and feasible in practice. On all these topics the comments of the experts and OSH professionals helped to improve the feasibility of the guideline.

## Conclusions

We developed a practice guideline to support occupational safety and health professionals in assessing the risk due to lifting and in selecting effective preventive measures for low back pain. This practice guideline is based on the best available scientific evidence and should be used by occupational safety and health professionals taking into account the values and preferences of workers and employers. Providing evidence-based guidance for risk assessment and lifting interventions will improve the quality of preventive practice and increase the impact of occupational health and safety advice. We strongly advise societies and associations of health professionals to support implementation of this guideline into daily practice by active education of their members in order to optimize successful health strategies to reduce the impact of lifting on low back pain among high risk groups of workers.

## Competing interests

The authors declare that they have no competing interests.

## Authors’ contributions

A core group of the project team consisting of JV, ML, AB and CH did the preparatory work on the basis of the project request of the Dutch Government and made a proposal for the clinical questions. These clinical questions were discussed with the other members of the project team consisting of PK, BV, LE, NVR, MVDW. Next, the core group performed the systematic literature search, performed the critical appraisal and wrote the evidence report draft, wrote the draft of the preliminary guideline and managed the external peer review and practice test of the preliminary guideline. All authors were involved in the revision of the draft guideline. PK drafted this manuscript. All authors were involved in reading and revision of the manuscript. All authors read and approved the final manuscript.
